# Case of Invasive Carcinoma Derived from Intraductal Papillary Mucinous Neoplasm Negative for GNAS Mutation

**DOI:** 10.7759/cureus.5940

**Published:** 2019-10-18

**Authors:** Nobuhiko Fukuba, Shunji Ishihara, Ichiro Moriyama, Yasunari Kawabata, Yoshitsugu Tajima

**Affiliations:** 1 Internal Medicine, Izumo City General Medical Center, Izumo, JPN; 2 Gastroenterology, Shimane University Hospital, Izumo, JPN; 3 Innovative Cancer Center, Shimane University Hospital, Izumo, JPN; 4 Digestive and General Surgery, Faculty of Medicine, Shimane University, Izumo, JPN

**Keywords:** gnas, intraductal papillary mucinous neoplasm, mucinous carcinoma, pancreas

## Abstract

A 70-year-old woman with loss of appetite was referred to our hospital for further examinations. Computed tomography revealed a low density tumor in the body of the pancreas measuring 4 cm in diameter. The main pancreatic duct was dilated on both the head and caudal side of the tumor. Magnetic resonance imaging showed the mass as a low intensity area in T1-weighted and high intensity area in T2-weighted images. Endoscopic retrograde cholangiopancreatography findings indicated that the main pancreatic duct was continuous with the lumen of the tumor. A cytological examination of pancreatic juice showed a class IV tumor. A distal pancreatectomy was performed as a curative resection procedure. The findings of hematoxylin eosin staining and mucus trait led to a diagnosis of invasive cancer derived from an intraductal papillary mucinous neoplasm (IPMN). We also performed sequencing analysis to investigate GNAS and K-RAS mutations in the tumor, though neither the GNAS mutation c602G>A nor K-RAS mutation c35G>A were observed. Cases negative for a GNAS mutation can be considered to have an increased risk of invasive cancer derived from an IPMN.

## Introduction

Recently, an intraductal papillary mucinous neoplasm (IPMN) is often found incidentally because of advancements in imaging diagnostic technologies such as magnetic resonance cholangiopancreatography and is one of the most frequently occurring pancreatic diseases. An IPMN can be classified as main pancreatic duct type and branched pancreatic duct type. Since the main pancreatic duct type has a high possibility of canceration, surgery in those cases should be considered [1]. On the other hand, a follow-up observation course is recommended for patients with the branch pancreatic ductal type without high-risk stigmata or other worrisome features. The most important consideration for follow-up observations of these cases is the potential of an invasive carcinoma derived from or concomitant with the IPMN [2]. Although the K-RAS mutation is known to play an important role in onset of normal type pancreatic duct cancer, the carcinogenic process of an invasive carcinoma derived from an IPMN remains unclear [3]. Particularly, at IPMN onset, a GNAS mutation has been reported to play a central role, though the relationship between GNAS mutation and invasive carcinoma derived from an IPMN has not been clarified.

Here, we report a case diagnosed as invasive carcinoma derived from an IPMN based on histopathological imaging and mucous trait findings, though was negative for both GNAS and K-RAS mutations. We consider the findings to be very interesting, as they infer the carcinogenic process of an IPMN-derived invasive carcinoma.

## Case presentation

**Figure 1:**
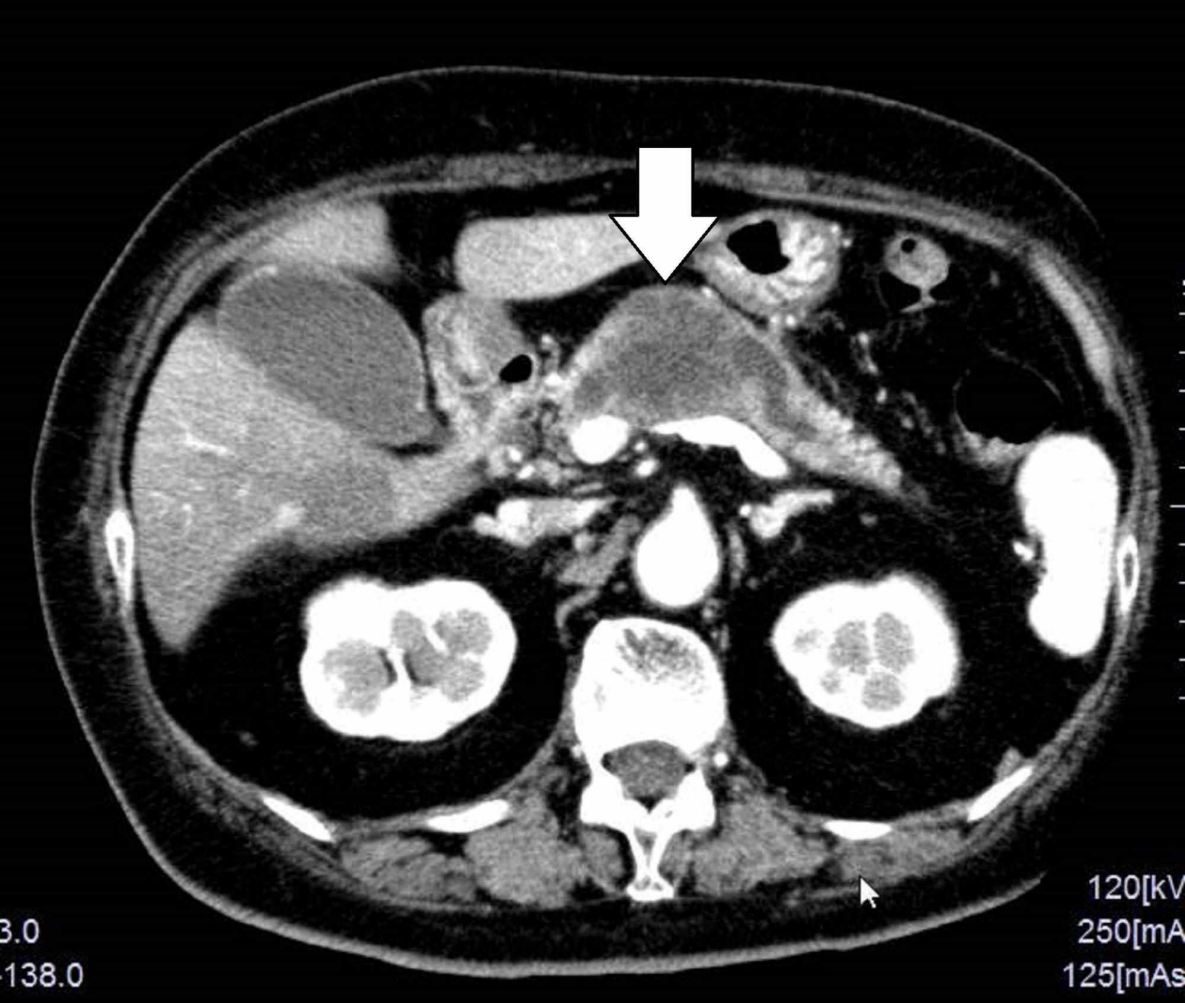
Dynamic enhanced computed tomography Dynamic enhanced computed tomography revealed a low density tumor in the body of the pancreas measuring 4 cm in diameter, with a poor contrast effect and unclear outline. The main pancreatic duct was dilated on both the head and caudal side of the tumor.

A 70-year-old woman with loss of appetite and weight loss of 10 kg over a three-month period consulted her family doctor. There was no pancreatic cancer patient in her family. A subsequent computed tomography (CT) scan showed a mass in the pancreas and she was referred to our hospital for further examinations. Laboratory results at admission were as follows: leukocyte count, 7.12 × 103/µL; platelet count, 300 × 103/L; total bilirubin, 1.3 mg/dL; aspartate aminotransferase, 19 U/L; alanine aminotransferase, 19 U/L; alkaline phosphatase, 216 U/L; amylase, 65 U/L; CEA, 171.9 ng/mL; CA19-9, 16 U/mL; DUPAN-2, <25 U/mL; and Span-1, 6 U/mL. Dynamic enhanced CT revealed a low density tumor measuring 4 cm in diameter in the body of the pancreas, with a poor contrast effect and an unclear outline (Figure 1).

**Figure 2:**
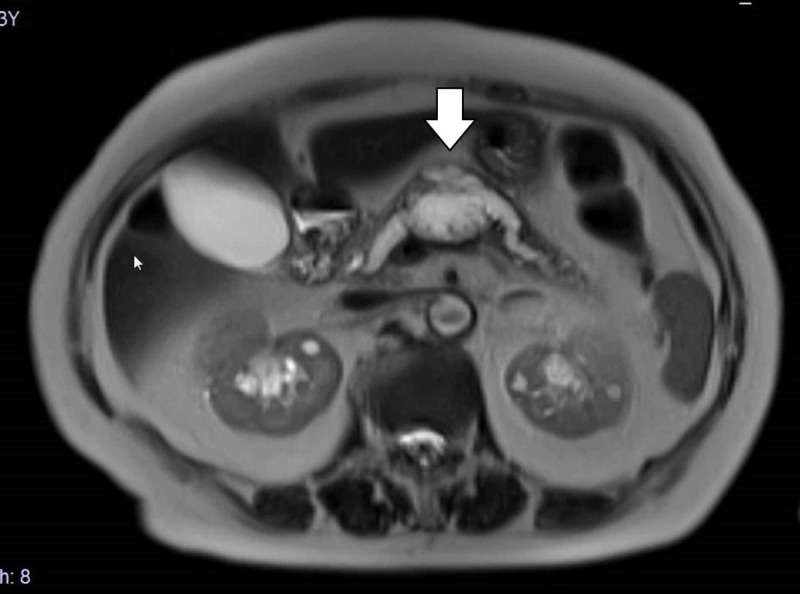
Magnetic resonance imaging T2-weighted magnetic resonance imaging showed the mass as a high intensity area.

The main pancreatic duct was dilated on both the head and caudal side of the tumor. T2-weighted magnetic resonance imaging (MRI) showed the mass as a high intensity area (Figure 2).

**Figure 3:**
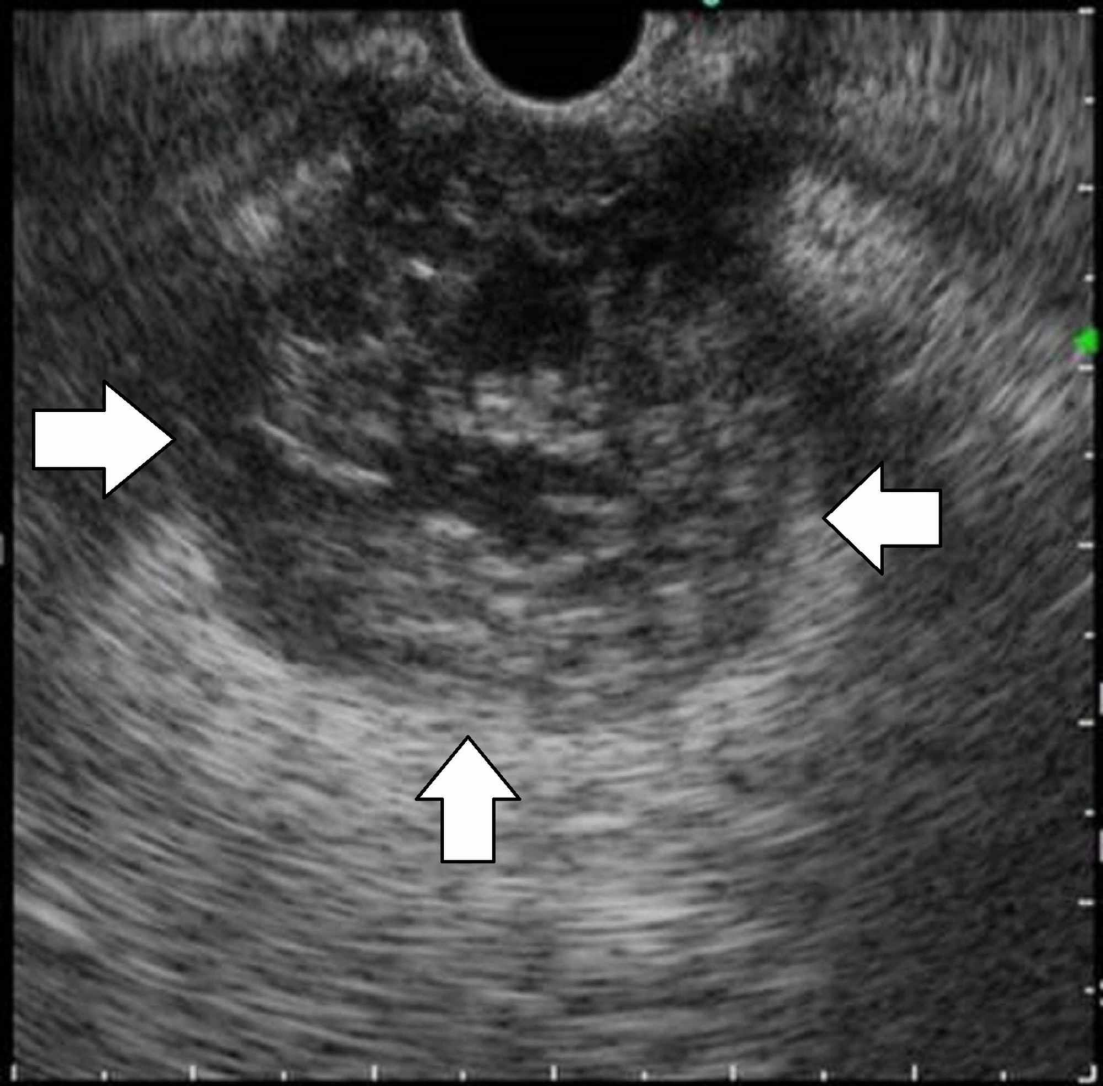
Endoscopic ultrasonography The internal echo of the tumor revealed a mosaic pattern in endoscopic ultrasonography findings.

**Figure 4:**
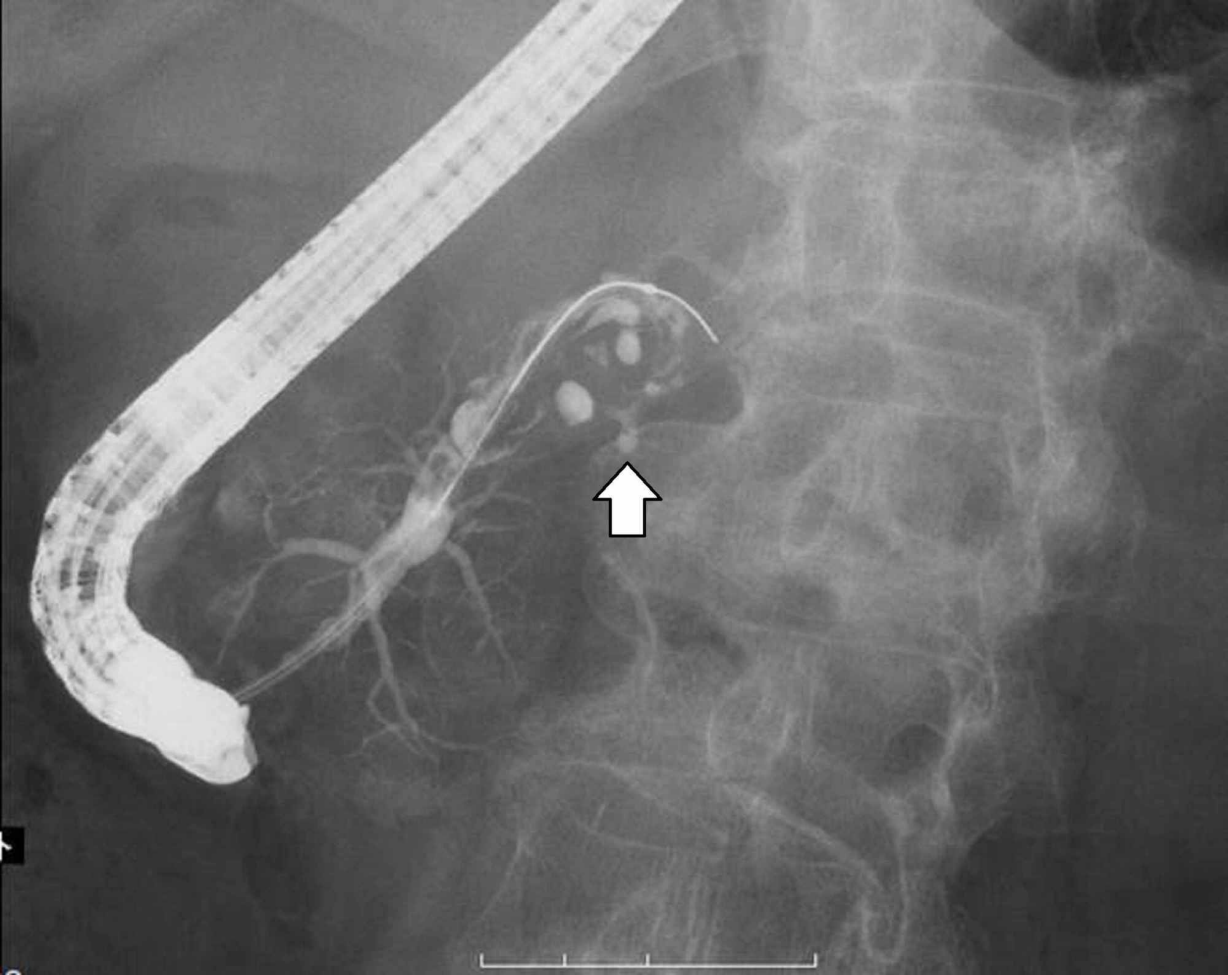
Endoscopic retrograde cholangiopancreatography The main pancreatic duct was continuous with the lumen of the tumor, with disruption and dilatation observed.

The internal echo of the tumor shown by endoscopic ultrasonography was a mosaic pattern (Figure 3), while endoscopic retrograde cholangiopancreatography (ERCP) indicated that the main pancreatic duct was continuous with the lumen of the tumor, with disruption and dilatation observed (Figure 4).

A cytological examination of pancreatic juice obtained from the patient during ERCP showed the tumor to be class IV. Differential diagnoses considered included invasive ductal carcinoma derived from an IPMN, mucinous carcinoma, anaplastic carcinoma, and mucinous cyst carcinoma. Neither distant nor lymph node metastasis was observed. We performed a pancreatic tail splenectomy as a curative resection procedure.

**Figure 5:**
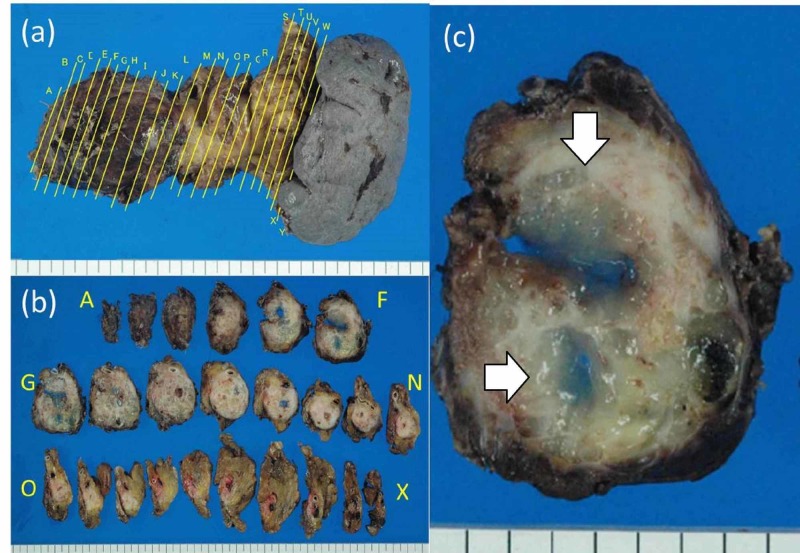
Macroscopic findings of resected specimen (a) A tumor 10 cm in size was found in the body of the pancreas, protruding to the ventral side. (b) The resected specimen was sliced. (c) Enlarged view of section G. On the cleaved surface, tumor lesions with mucus in the lumen were found.

Macroscopic findings of the resected specimen showed a tumor 10 cm in size in the body of the pancreas and protruding to the ventral side. On the cleaved surface, tumor lesions with mucus in the lumen were recognized, and occupied most of the body tail (Figure 5).

**Figure 6:**
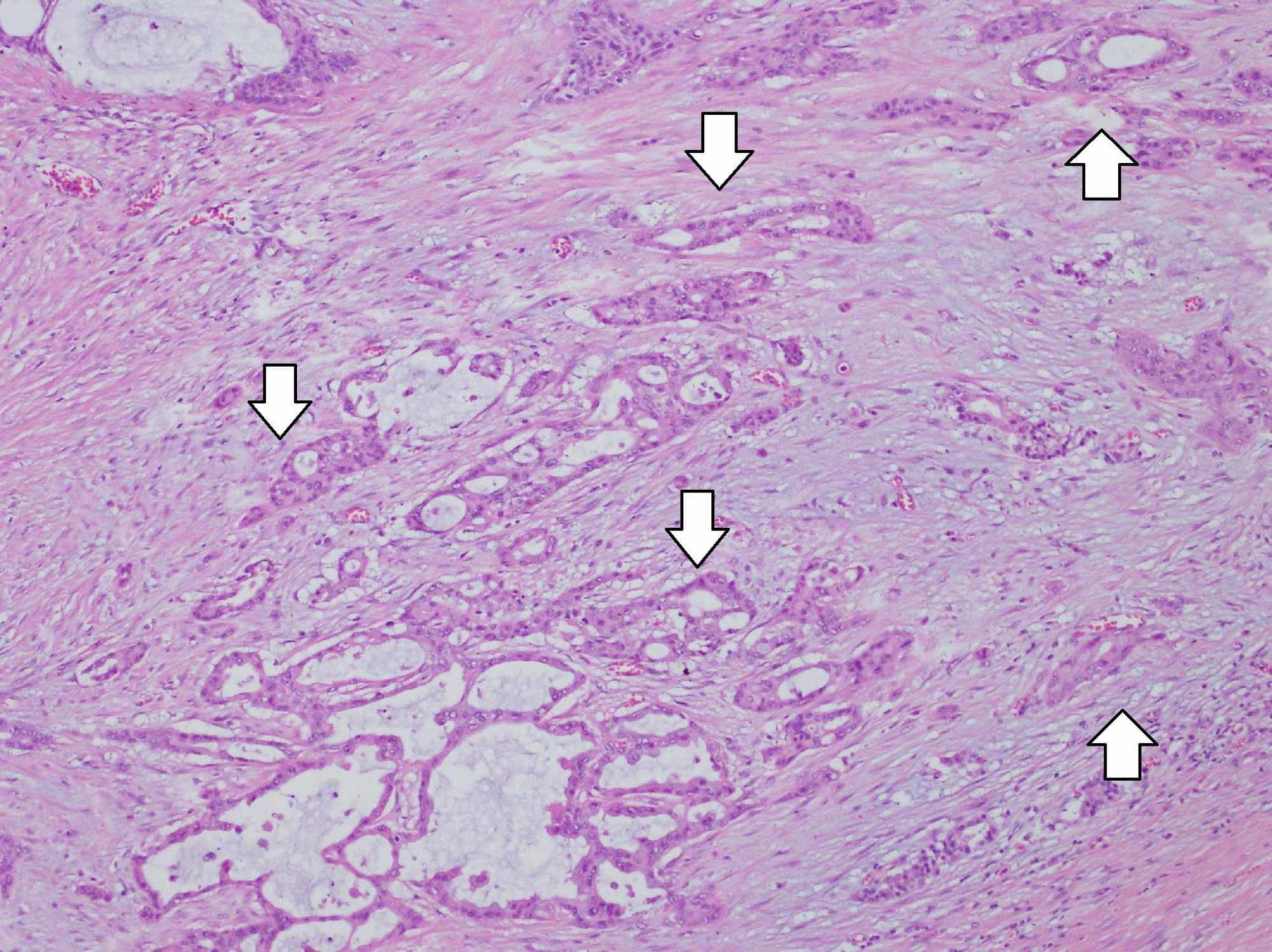
The invasive part of the tumor Most of the macroscopic mass corresponded to the invasive part, in which tumor cells with mucous and nuclear atypia showed increasing invasion with construction of duct structures (arrows). Hematoxylin eosin staining.

Histologically, the tumor was divided into invasive and non-invasive parts. Most of the macroscopic mass corresponded to the invasive part, in which tumor cells with mucous and nuclear atypia showed increasing invasion of the constructing duct structures and mucus stored in the tumor duct (Figure 6).

**Figure 7:**
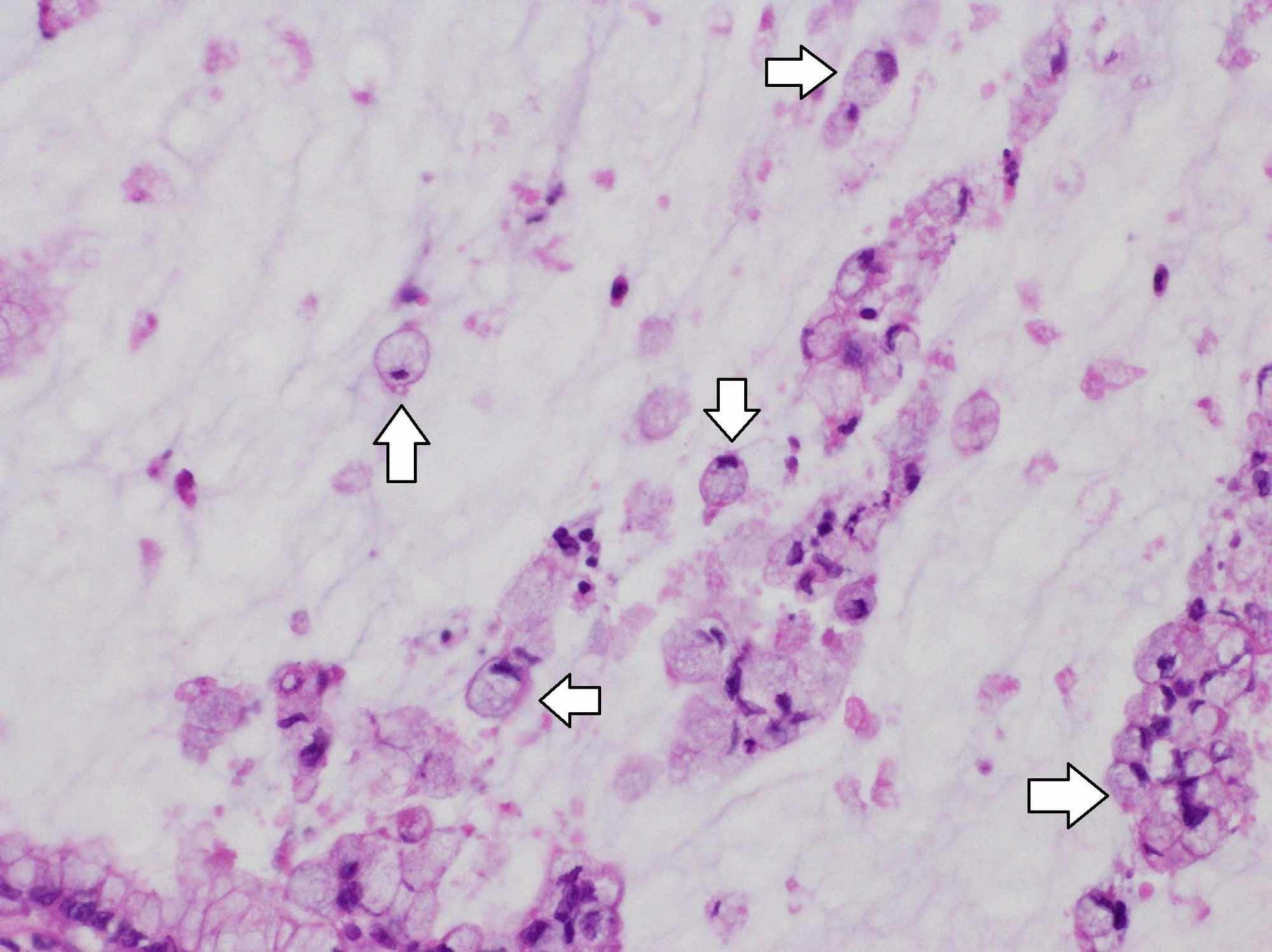
Signet ring cells Signet ring cells were found suspended in mucus (arrows). Hematoxylin eosin staining.

Furthermore, numerous signet ring cells were seen suspended in mucus (Figure 7).

**Figure 8:**
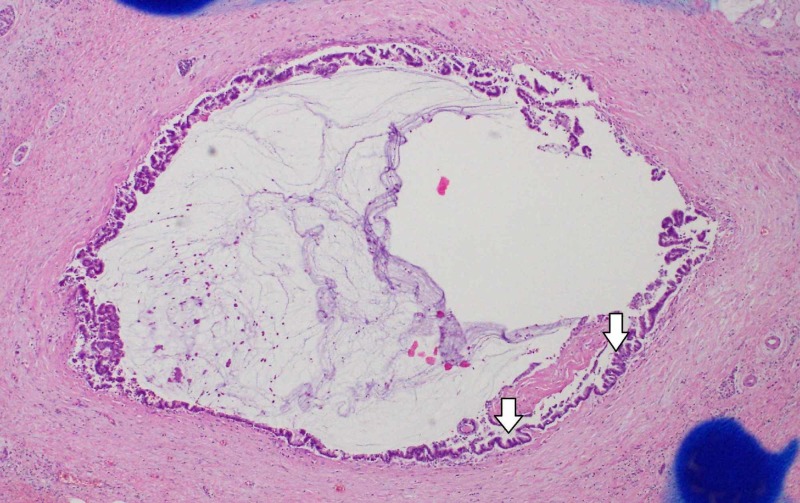
The non-invasive part of the tumor In the non-invasive part, the papillary epithelium of the main pancreatic duct had spread to the head and caudal side of the main lesion, and was continuous with the main lesion. Hematoxylin eosin staining.

On the other hand, in the non-invasive part, papillary epithelium of the main pancreatic duct had spread to both the head and caudal side of the tumor, and was continuous with the main lesion (Figure 8).

**Figure 9:**
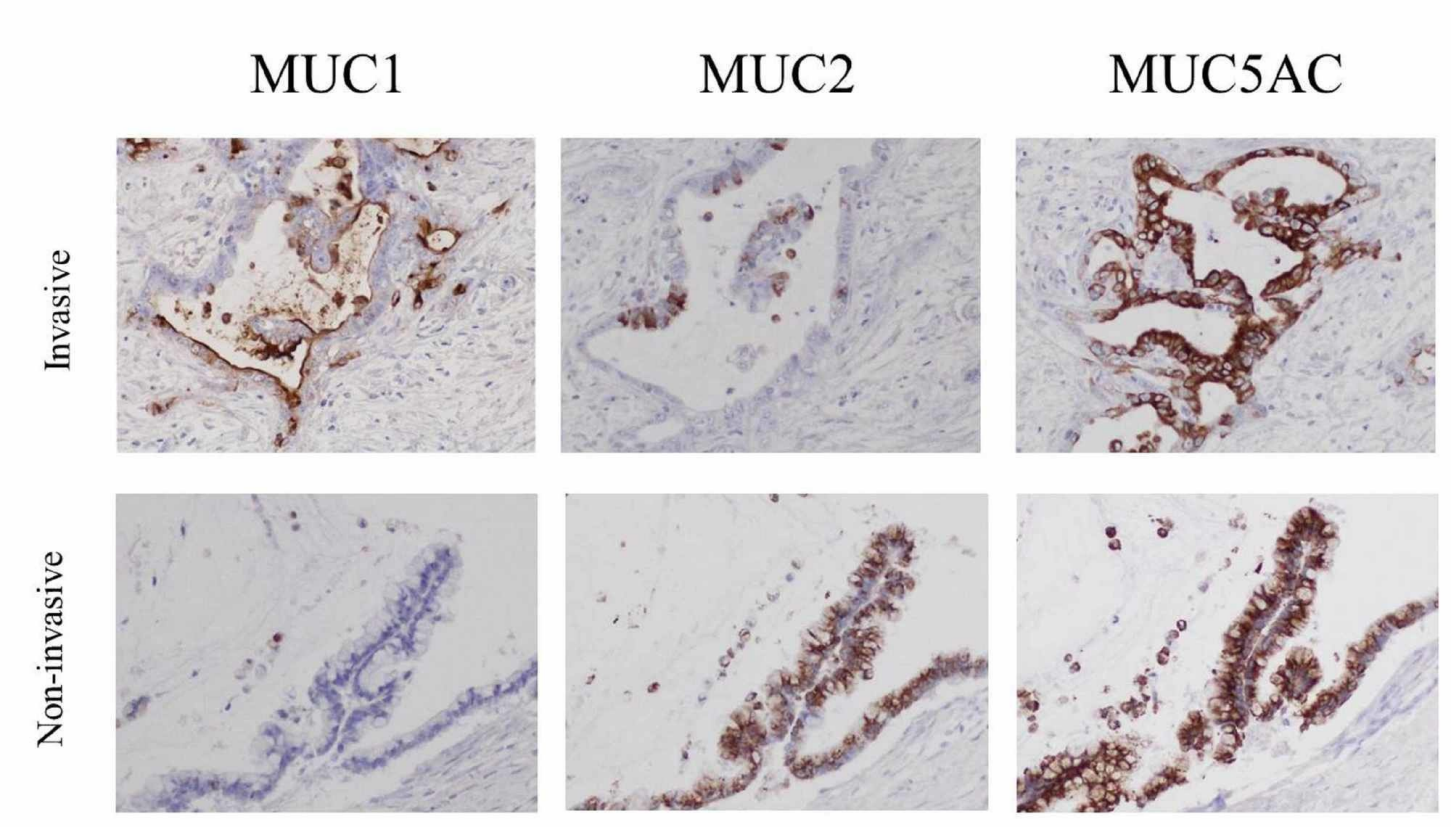
Immunostaining for MUC1, MUC2, and MUC5AC The upper images show the invasive part and the lower images the non-invasive part of the tumor. The invasive part was positive for all three types of mucin, while the non-invasive part was positive for only MUC2 and MUC5AC.

Pathological features of mucinous cyst neoplasm such as ovarian-type stroma were not observed. Results of hematoxylin eosin staining of the resected tumor excluded a mucinous cyst carcinoma, and the diagnosis was IPMN-derived invasive cancer or mucinous carcinoma. To distinguish between those, immunostaining for MUC1, MUC2, and MUC5AC was performed. The invasive part was positive for all three types of mucin, while the non-invasive part was positive for only MUC2 and MUC5AC, which supported IPMN-derived invasive cancer (Figure 9).

**Figure 10:**
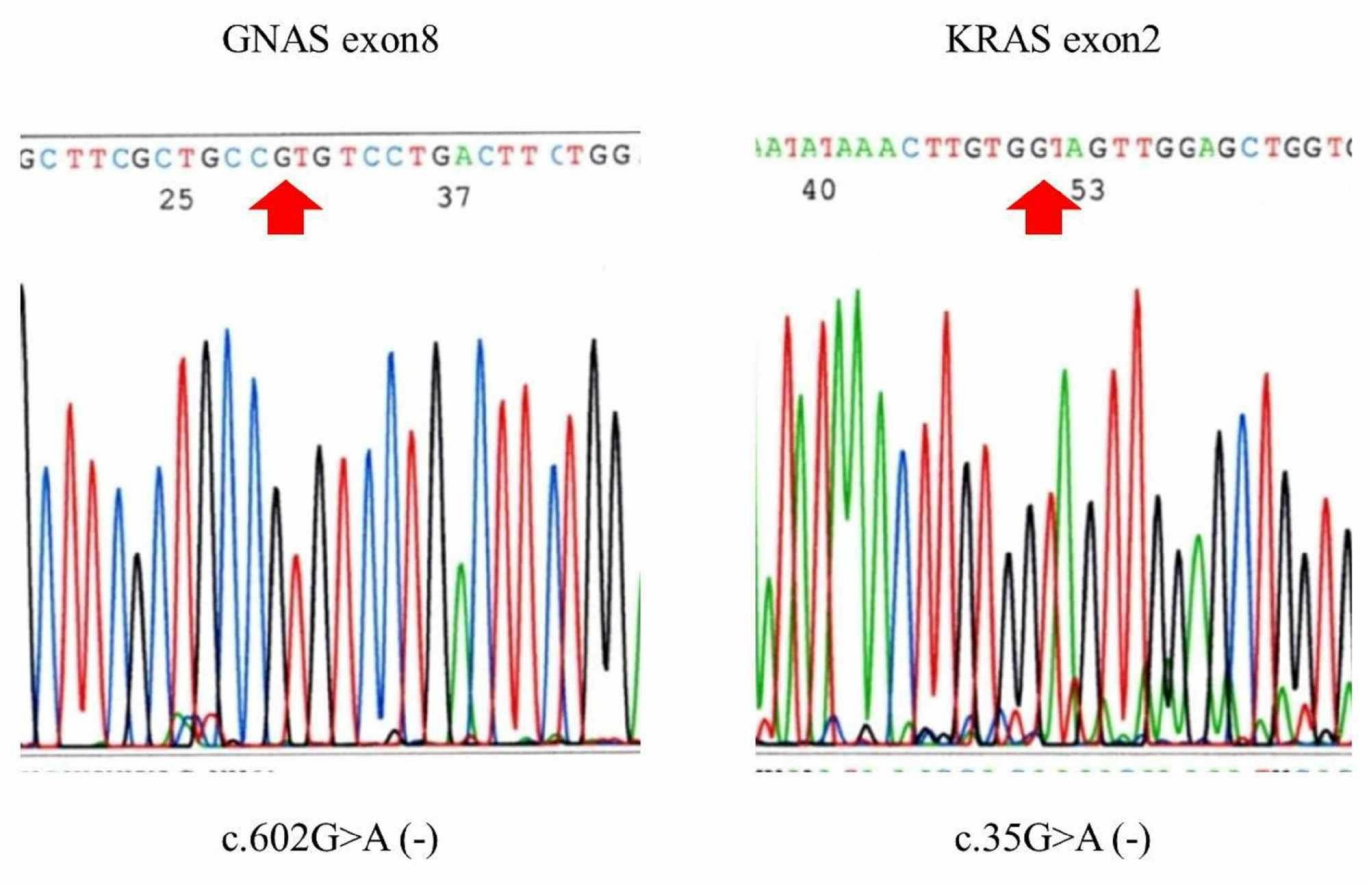
Results of sequencing of the GNAS and KRAS genes Neither the GNAS mutation c602G>A nor KRAS mutation c35G>A was observed.

Additionally, we examined for GNAS and K-RAS mutations in the tumor. The main lesion was scraped to obtain a representative section, which was embedded in paraffin, with DNA isolated using a Takara DEXPAT kit (Takara Bio). PCR amplification of the GNAS gene exon 8 and K-RAS gene exon 2 regions was performed using the isolated DNA. The forward and reverse primers used for the GNAS gene were 5’-TTATTACTGTTTCGGTTGGC-3’ and 5’-TCAAGAAACCATGATCTCTG-3’, respectively, and those for the K-RAS gene were 5’-CATGTTCTAATATAGTCACATTTTC-3’ and 5’-ATCGTCAAGGCACTCTTGCC -3’, respectively [4]. Amplifications were performed by initial denaturation at 94°C for 1 minute, followed by 30 cycles of denaturation at 98°C for 10 seconds, annealing at 61°C for 15 seconds, and extension at 68°C for 30 seconds using Takara Gflex polymerase (Takara Bio). The PCR products thus obtained were then purified using an Illustra ExoProStar kit (GE Healthcare), and sequenced by use of a Big Dye cyclic sequencing kit and ABI 310 sequencer (Applied Biosystems, Forster City, CA). Sequencing analysis was successfully performed. Neither the GNAS mutation c602G>A nor K-RAS mutation c35G>A was observed (Figure 10).

## Discussion

There are a variety of pancreatic cystic diseases, including IPMN, mucinous cyst neoplasm, serous cyst neoplasm, and solid pseudopapillary neoplasm, of which IPMN is the most frequent. Most affected patients have a good prognosis, though some cases may have high risk stigmata or worrisome features, thus surgery should be considered [1, 5]. On the other hand, cases of invasive carcinoma derived from or concomitant with an IPMN have been reported [2], which are distinguished by the positional relationship with the IPMN, and it is presumed that the related carcinogenesis mechanisms are also different. In the present case, the invasive part tissue type was mucinous carcinoma, and the mucous trait of the infiltrated area was positive for both MUC2 and MUC5AC. According to the report of Yamaguchi et al., 24.6% of invasive carcinoma tumors derived from an IPMN were found to be mucinous carcinoma, of which 1.3% were normal type pancreatic cancer and 0% invasive carcinoma concomitant with an IPMN [2]. Thus, we considered that our case was likely to be an IPMN-derived invasive carcinoma. However, the features of cases with carcinogenesis of an invasive carcinoma derived from or concomitant with an IPMN remain to be elucidated.

An invasive carcinoma derived from an IPMN is considered to have a different carcinogenic process as compared to a normal type invasive carcinoma. For cases with normal type pancreatic cancer, K-RAS,p53,CDKN2A, and SMAD4 genetic abnormalities have been noted, though no specific genetic abnormality has been reported for invasive cancer derived from an IPMN [6]. We diagnosed the present case as invasive IPMN-derived carcinoma based on tissue type and mucinous trait, and speculate that genetic abnormality characteristics related to IPMN development remain to be revealed.

In recent years, the GNAS mutation has attracted attention as a representative gene mutation associated with IPMN [4, 7-10]. Furukawa et al. analyzed 118 cases of IPMN and reported that a GNAS mutation was present in 40.7%, which led to their conclusion that GNAS mutations are common and specific for an IPMN, and activation of G-protein signaling appears to play a pivotal role in its occurrence [4]. However, the present case was negative for a GNAS gene mutation. That finding is extremely interesting, as the diagnosis was invasive carcinoma derived from IPMN. Although a GNAS gene mutation is known to be part of the cause of IPMN, it is possible that other genetic abnormalities may be in the background of cases negative for that mutation. Notably, cases of IPMN associated with McCune-Albright syndrome due to the GNAS gene mutation have been reported to have a lower level of canceration [11]. Although several reports have indicated genes causative of IPMN, cases that are negative for the GNAS mutation can be considered to have increased risk of invasive cancer.

## Conclusions

We experienced a case of invasive cancer derived from IPMN without GNAS and K-RAS mutations. This is a valuable case for examining the genetic background of the development of invasive cancer derived from IPMN.
